# *ANXA1* and *ARG2* drive T cell proliferation in ischemia-reperfusion injury: integrated bulk and single-cell transcriptomic analysis

**DOI:** 10.3389/fcell.2025.1673163

**Published:** 2025-09-04

**Authors:** Haofeng Zheng, Kaiming He, Jianchao Wei, Wangtianxu Zhou, Zhiyi Kong, Qingfu Dai, Jieyi Dong, Zihuan Luo, Qiquan Sun

**Affiliations:** Department of Renal Transplantation, Guangdong Provincial People’s Hospital (Guangdong Academy of Medical Sciences), Southern Medical University, Guangzhou, China

**Keywords:** ischemia-reperfusion injury, T cell proliferation, *ANXA1*, *ARG2*, scRNA-sequencing

## Abstract

Ischemia-reperfusion injury (IRI) represents a common pathophysiological condition and serves as a shared mechanism underlying diverse critical diseases, including acute kidney injury, myocardial infarction, and stroke. T cells are increasingly recognized as central mediators of immune responses during IRI; however, the mechanisms governing their proliferation remain poorly characterized. Herein, an integrative analysis of bulk and single-cell transcriptomic datasets across multiple organ models was performed to investigate the role of T cell proliferation–related genes in IRI. We identified *ANXA1* and *ARG2* as key IRI-associated genes, both of which exhibited consistent upregulation during the early stages of injury. Immune infiltration analysis demonstrated that *ANXA1* expression correlated most strongly with central memory CD4^+^ T cell infiltration, whereas *ARG2* was linked to T helper 17 cell infiltration. Drug prediction and molecular dynamics simulation further identified Hydrocortamate and NS6180 as potential therapeutic agents targeting T cell proliferation. Single-cell RNA sequencing not only confirmed the active involvement of T cells in IRI progression but also highlighted *ANXA1* as a particularly prominent regulator. A renal IRI model was also used to further confirm altered T cell activity and differential expression of these key genes *in vivo*. Collectively, these findings elucidate the molecular mechanisms driving T cell proliferation in IRI, positioning *ANXA1* and *ARG2* as promising pan-organ IRI biomarkers and therapeutic targets for mitigating tissue damage and promoting repair.

## 1 Introduction

Ischemia-reperfusion injury (IRI) represents a vital pathophysiological mechanism that paradoxically intensifies tissue damage and organ dysfunction once blood flow is reestablished after periods of ischemia or hypoxia (Zhang M. et al., 2024). As a shared mechanism among various severe conditions, including acute kidney injury (AKI), myocardial infarction, and ischemic stroke, IRI substantially contributes to the global healthcare burden (Hoste et al., 2018; Saini et al., 2021; Heusch, 2024). Renal ischemia-reperfusion injury (RIRI), a primary driver of AKI, frequently occurs in clinical scenarios including kidney transplantation, cardiac surgery, trauma, and partial nephrectomy (Zhang et al., 2020). Specifically, RIRI is an unavoidable outcome of kidney transplantation and is highly associated with delayed graft function, acute rejection, and loss of the graft (Zhang et al., 2024a). Despite extensive research into the mechanisms and treatments of IRI, effective therapeutic strategies remain limited (Zhang M. et al., 2024). Elucidating the key mechanisms of IRI, identifying specific druggable targets, and developing specific interventions to mitigate IRI progression are critical for improving organ function across various pathological conditions.

IRI is characterized by robust inflammation in injured tissues, marked by extensive immune cell infiltration and inflammation-mediated tissue damage, creating a complex immune microenvironment (Zhang M. et al., 2024). Although IRI is predominantly derived by innate immunity in the early immune response (Zheng et al., 2021; Zhang et al., 2024c), recent findings underscore the crucial role of T cells, which are key elements of adaptive immunity and flexible regulators in immune responses. (Sun et al., 2023), in exacerbating tissue injury (Huang et al., 2007; Göcze et al., 2018; Lee and Jang, 2022). Rapid T cell infiltration and proliferation occur early, and T cell depletion has been shown to attenuate damage and enhance functional recovery (Dellepiane et al., 2020). However, the mechanisms underlying rapid T cell activation remain poorly understood, despite their growing recognition as pivotal contributors to IRI progression.

Clarifying the regulatory mechanisms of T cell proliferation in IRI and identifying potential intervention targets may provide new directions for diagnosis and therapy. Emerging omics approaches enable the identification of gene sets associated with specific functional phenotypes (Jeon et al., 2024), offering new avenues to elucidate T cell activity in IRI. Although T cell proliferation-related genes (TRGs) had been well defined in several studies (Huang et al., 2022; Legut et al., 2022; Cui et al., 2023; Hai et al., 2024), their specific roles in IRI remain undefined. Bulk RNA sequencing reveals tissue-specific gene expression changes in diseased states, while single-cell RNA (ScRNA) sequencing captures gene expression at the single-cell level, revealing cellular heterogeneity and intercellular interactions (Li and Wang, 2021). Integrative bioinformatics approaches linking TRGs to IRI could provide novel insights for diagnosis and treatment.

To investigate the involvement of T cells in IRI, this study integrates multiple transcriptomic approaches to systematically characterize the dynamic expression landscape of TRGs, identify key regulatory genes, and explore their underlying mechanisms and therapeutic potential in RIRI. Following key gene identification, their expressions were validated in independent datasets derived from heart IRI (HIRI) and brain IRI (BIRI) model to assess their pan-organ consistency. Finally, *in vivo* experiments based on RIRI model were conducted to confirm the biological relevance of candidate genes. This study offers novel insights into T cell-mediated immune responses in IRI and provides a basis for the discovery of new therapeutic targets for IRI-induced tissue injury and repair.

## 2 Materials and methods

### 2.1 Data collection

All datasets were obtained from the Gene Expression Omnibus (GEO) database. (https://www.ncbi.nlm.nih.gov/geo/). The GSE98622 dataset (Liu et al., 2017), as the exploration set, consists of six samples in sham group, including three samples at 4 and 24 h respectively, and 24 kidney RIRI samples at different time points after ischemia-reperfusion, including three samples at 2 h, 4 h, 24 h, 48 h, 72 h, 7 days, 14 days, 28 days, and 12 months respectively. The GSE267650 dataset (Heruye et al., 2024), an external renal validation set, consists of five control kidney tissue samples in sham group and 36 kidney tissue samples at different time points after ischemia-reperfusion, including five samples at 20 min, 4 h, 16 h, 24 h, 36 h, 48 h respectively, and six samples at 72 h, in RIRI group. The GSE131193 dataset was used as validation of BIRI (Kestner et al., 2020), consisting of 12 control brain tissue samples at different time points after sham surgery, including six samples at 1 and 7 days respectively, 12 brain tissue samples at different time points after IRI, including six samples at 1 and 7 days respectively in BIRI group. The GSE160516 dataset was used as validation of HIRI (Zhang and Li, 2020), consisting of four control heart tissue samples and 12 heart tissue samples at different time points after IRI, including four samples at 6 h, 24 h, and 72 h. The GSE139506 dataset (Rudman-Melnick et al., 2020), a single-cell dataset, consisted of one control kidney tissue sample and nine kidney tissue samples at different time points after ischemia-reperfusion, including one sample at 1 day, 2 days, and 4 days, and two samples at 7 days, 11 days, and 14 days. Replicate samples at 7 days, 11 days, and 14 days were merged, with each time point consolidated into a single dataset.

A total of 216 mouse genes related to T cell proliferation were obtained. Specifically, gene sets related to T cell proliferation were first integrated from previous studies (Huang et al., 2022; Legut et al., 2022; Cui et al., 2023; Hai et al., 2024). After deduplication, 211 TRGs were identified. Subsequently, human gene symbols were converted to mouse gene symbols using gProfiler (https://biit.cs.ut.ee/gprofiler/gost), with gene aliases retained for consistency. Finally, a total of 216 TRGs were obtained (Supplementary Table S1).

### 2.2 Temporal expression analysis and identification of candidate genes

The Mfuzz algorithm in the “ClusterGVis” (v 0.1.2) (https://github.com/junjunlab/ClusterGVis) was used for clustering analysis of expression patterns in the transcriptome data at different time points of the training set, to identify potential time-series expression profiles. The Mfuzz method is based on Fuzzy C-Means Clustering (FCM). The expected number of clusters was set to 10, and genes with similar expression patterns across 10 time-series groups (Sham samples and IRI samples at 2 h, 4 h, 24 h, 48 h, 72 h, 7 days, 14 days, 28 days, and 12 months) were clustered.

To identify genes involved in RIRI pathogenesis, the “limma” (v 3.56.2) was employed for differential expression analysis between the RIRI and Sham groups of the exploration dataset (Ritchie et al., 2015), with the criteria of both p < 0.05 and |log2 Fold Change (FC)| >1. Volcano plots and heatmaps were generated to visualize the up- and downregulated genes, ranked by |log2FC| from highest to lowest, using “ggplot2” (v 3.5.1) and “ComplexHeatmap” (v 2.16.0) (Gu et al., 2016; Wickham, 2016).

In this study, we focus on the early T cell reaction in IRI, so gene clusters showing upregulated expression trends in the early stage were selected as important genes for subsequent analysis. The intersection of differentially expressed genes (DEGs) in RIRI and upregulated TRGs was then identified using “ggvenn” (v 0.1.10) (https://github.com/yanlinlin82/ggvenn), and these genes were designated as candidate genes.

Enrichment analysis included the Kyoto Encyclopedia of Genes and Genomes (KEGG) and Gene Ontology (GO). GO covers biological processes (BP), molecular functions (MF), and cellular components (CC). These analyses were performed on candidate genes using “clusterProfiler” (v 4.8.3) (Xu et al., 2024; Yu, 2024). Visualization was done for the top 5 GO terms and the top 10 KEGG pathways.

### 2.3 Identification and validation of key genes

To identify key genes among the candidate genes, protein–protein interaction (PPI) network of the candidate genes coded proteins was constructed by the STRING database (https://string-db.org/) with the confidence score threshold set at >0.15. Then the network was further analyzed by CytoHubba, a plug-in in Cytoscape, to evaluate node centrality and identify hub genes (Shannon et al., 2003; Cline et al., 2007; Chin et al., 2014). Five independent algorithms, including MNC, DMNC, Degree, EcCentricity, and Radiality, were employed to rank nodes to ensure the robustness of hub gene selection. The top five ranked genes were identified for each algorithm, and the intersection of these five gene sets was defined as the final set of core genes used for subsequent analyses.

Subsequent analysis involved assessing the differential expression of significant genes between the RIRI and Sham groups using the Wilcoxon test (p < 0.05) in the exploration and validation datasets. Genes exhibiting consistent expression trends and significant differences in both datasets were confirmed as key genes. Using the ‘ggplot2’ package (v3.5.1) in R, line graphs were produced to visualize temporal expression dynamics, displaying key gene expression across eight time intervals (Sham, 20 min, 4 h, 16 h, 24 h, 36 h, 48 h, and 72 h post-IRI) in the validation dataset. To evaluate the consistency of key gene expression across different organs in IRI, their expression levels and dynamics were also validated in HIRI and BIRI datasets.

### 2.4 Gene set enrichment analysis (GSEA)

Key genes in the exploration dataset were analyzed using GSEA to understand their biological functions in the development of RIRI, utilizing the ‘c2.cp.kegg.symbols.gmt’ reference gene set from the Molecular Signatures Database (https://www.gsea-msigdb.org/gsea/msigdb) was used. Correlation coefficients between key genes and other gene datasets were calculated using the “psych” package (v2.4.6.26; Revelle, 2024), with genes ranked in descending order of correlation. GSEA was then conducted using the “clusterProfiler” package (v4.8.3) with significance thresholds of p.adjust <0.05 and q-value <0.25 (Xu et al., 2024; Yu, 2024). Using the ‘enrichplot’ package (v1.20.3), the top five pathways enriched for each key gene were visualized, sorted by the absolute normalized enrichment score (|NES|) (https://github.com/YuLab-SMU/enrichplot).

### 2.5 Immunoinfiltration analysis

The “GSVA” package (v1.53.28) was used to evaluate the infiltration levels of 28 immune cell types across all samples in the exploration dataset (Hänzelmann et al., 2013). A heatmap visualizing these infiltration levels was generated using the “pheatmap” package (v1.0.12, https://github.com/raivokolde/pheatmap). Differences in infiltration levels of the 28 immune cell types between the RIRI and Sham groups were assessed using the Wilcoxon test. A box plot illustrating these differences was created using the “ggplot2” package (v3.5.1). Immune cell types showing significant differences were designated as differential immune cells. Relationships among differential immune cells and between these cells and key genes were analyzed with correlations considered significant at |correlation coefficient| >0.3 and p < 0.05. Correlation heatmaps were visualized using the “ggcorrplot” package (v0.1.4.1).

### 2.6 Drug prediction and molecular docking

To explore the relationships between key genes and potential therapeutic drugs in IRI, drug-gene interactions were retrieved from the Drug-Gene Interaction Database (DGIdb; https://www.dgidb.org/) and visualized using Cytoscape (v3.10.3). For each significant gene, a small-molecule drug known to inhibit cell proliferation was selected for molecular docking analysis. The Protein Data Bank was used to acquire the protein structures of the essential genes. (https://www.rcsb.org/), and the corresponding drug structures were retrieved in SDF format from the PubChem database (https://pubchem.ncbi.nlm.nih.gov/). Molecular docking was performed by uploading protein and drug structures to the CB-Dock2 online platform (https://cadd.labshare.cn/cb-dock/php/blinddock.php), where binding free energies were calculated to assess interaction affinities.

### 2.7 Molecular dynamics simulation (MDS)

To investigate the interaction strength and stability between drugs and receptor proteins corresponding to key genes in RIRI, MDS were performed using GROMACS software (v2024.4) with the AMBER99SB-ILDN force field (Pronk et al., 2013). The simulation system utilized the TIP3P water model within a cubic simulation box, maintaining a 1 nm distance between the protein and box edges. Ions were added to ensure electrical neutrality. Energy minimization was conducted using the steepest descent algorithm, followed by NVT (constant number of particles, volume, and temperature) and NPT (constant number of particles, pressure, and temperature) ensemble simulations. Temperature coupling was accomplished using the V-rescale technique at 300 K, employing a 2 fs time step and lasting 100 ps for the NVT and NPT phases. MDS was then run for 20 ns. Key metrics, including root-mean-square deviation (RMSD), root-mean-square fluctuation (RMSF), total energy, and hydrogen bond counts, were analyzed. Lower RMSD and RMSF fluctuations, reduced total energy, and a higher number of hydrogen bonds indicated stronger and more stable drug-protein binding interactions.

### 2.8 The ScRNA sequencing data processing

The GSE139506 dataset’s ScRNA sequencing data were combined using the ‘CreateSeuratObject’ function from the ‘Seurat’ package (version 5.1.0) (Hao et al., 2024). Data were processed and filtered to retain: (1) genes expressed in >3 cells; (2) cells with 200–4,000 expressed genes; (3) cells with <15% mitochondrial gene content; and (4) cells with total gene expression counts between 200 and 10,000. Normalized data were obtained using the “LogNormalize” method via the “NormalizeData” function. The ‘vst’ method of the ‘FindVariableFeatures’ function was used to identify highly variable genes (HVGs). The data was then scaled using the ‘ScaleData’ function, and principal component analysis (PCA) was performed. The “ElbowPlot” function determined significant principal components up to the inflection point for downstream analysis. Using the ‘IntegrateLayers’ function, batch effects were adjusted with the ‘Harmony’ algorithm. Unsupervised clustering was performed using the “FindNeighbors” and “FindClusters” functions (resolution = 1), with cell clusters visualized via uniform manifold approximation and projection (UMAP) using the “RunUMAP” function. Cluster-specific marker genes were identified using the “FindAllMarkers” function (logfc.threshold = 0.25, min. pct = 0.25, test. use = “auc”). Cell types were annotated by comparing cluster-specific genes with literature-reported marker genes (Rudman-Melnick et al., 2020) and using the “SingleR” package (v2.2.0) (Aran et al., 2019). Marker gene expression intensities were visualized in a bubble plot.

### 2.9 Identification of key cells

As the study focused on T cell changes during RIRI occurrence, T cells were selected as the key cells for subsequent analysis. First, in all samples from the GSE139506 dataset, the “DimPlot” function from “Seurat” (v 5.1.0) was employed to generate UMAP plots to display the distribution of T cells across different time points. Subsequently, the distribution maps showing the expression of key genes in T cells across various time points were created using the “FeaturePlot” function.

### 2.10 Cell communication and pseudotime analysis

The ‘CellChat’ package (v1.6.1) was used to analyze intercellular communication networks among annotated cell types in the GSE139506 dataset (Jin et al., 2021). Ligand-receptor (LR) pairing patterns were examined to infer potential intercellular interactions, with significance thresholds set at p < 0.05 and log2mean (Molecules 1 and 2) ≥ 0.1. Communication patterns between T cells and other cell types were compared between RIRI and Sham samples. To investigate dynamic expression patterns and temporal trajectories of key genes in T cell subtypes during RIRI, secondary dimensionality reduction and clustering utilized T cell subtype marker genes from mouse kidney tissue as recorded in the CellMarker database (http://www.bio-bigdata.center/index.html). Re-clustered T cells were annotated into distinct subpopulations. Using UMAP with default settings in the ‘monocle3’ package (v1.3.1), these subpopulations were mapped into a low-dimensional space (Cao et al., 2019). A root node was selected to order T cell subpopulations and visualize their developmental temporal sequence. Changes in T cell subtypes across different time points were further visualized with “monocle3” (v1.3.1).

### 2.11 Experimental animals and IRI-induced model

Subsequent experiments utilized male C57BL/6 mice aged 6–8 weeks, with all procedures conducted following China’s animal welfare guidelines (Laboratory Animal Guidelines for Ethical Review of Animal Welfare, GB/T 35892-2018) and approved (KY-Z-2022-026-02) by the Animal Care and Use Committee of Guangdong Province People’s Hospital (Guangdong, China). Mice were randomly allocated either to the IRI or Sham group (*n* = 5 per group). And the IRI was induced in mice based on previously reported methods (Zheng et al., 2021). The bilateral IRI model was created by clamping both renal pedicles for 26 min, while keeping the body temperature stable between 36.5 °C and 37 °C in the IRI group. The mice were euthanized 24 h after reperfusion. Samples from the kidneys and blood were obtained for later examination.

### 2.12 Measurement of renal function and renal histology

Blood samples underwent centrifugation at 2,000 × g for 10 min at 4 °C, and subsequently at 8,000 × g for 10 min at 4 °C. The serum was extracted and kept frozen at −80 °C until it was required. An automatic biochemistry analyzer (7020; Hitachi, Tokyo, Japan) was used to measure creatine (Cr) and blood urea nitrogen (BUN) levels. Renal tissues were collected without perfusion and preserved in 4% paraformaldehyde. Sections of kidney paraffin, measuring 4 μm, were stained with Hematoxylin and Eosin (H&E) and Periodic Acid-Schiff (PAS) to evaluate kidney damage.

### 2.13 Flow cytometry

The proliferation of T cells was analyzed by flow cytometry. Anti-Mouse CD16/CD32 (553141; RRID: AB_394656, clone 2.4G2; BD Biosciences, San Jose, CA, United States) was used for nonspecific Fc block. BV421 anti-mouse CD45 (30-F11; RRID: AB_2562559; BioLegend, San Diego, CA, United States), PC/Cyanine7 anti-mouse CD3 (17A2; RRID: AB_2242784; BioLegend, San Diego, CA, United States), FITC anti-mouse CD4 (H129.19, RRID: AB_1279237; BioLegend, San Diego, CA, United States), and APC anti-mouse CD8a (53-6.7; RRID: AB_312751; BioLegend, San Diego, CA, United States) were used for flow cytometry. A FACS Calibur cytometer by Becton Dickinson (BD) in Bedford, MA, United States, was used to gather data, which was then analyzed with FlowJo software from Tree Star in Ashland, OR, United States.

### 2.14 Real-time quantitative PCR

According to the manufacturer’s guidelines, fresh tissues were homogenized, and total RNA was isolated using the RNAeasy™ Animal RNA Isolation Kit with Spin Column (R0024; Beyotime, Shanghai, China). To calculate mean fold changes, the average of three duplicate measurements was normalized to Gapdh, utilizing the 2−△△CT method. The sequences for the primer pairs are provided in Supplementary Table S2.

## 3 Results

### 3.1 Temporal dynamics of TRG expression and candidate genes identification

**FIGURE 1:**
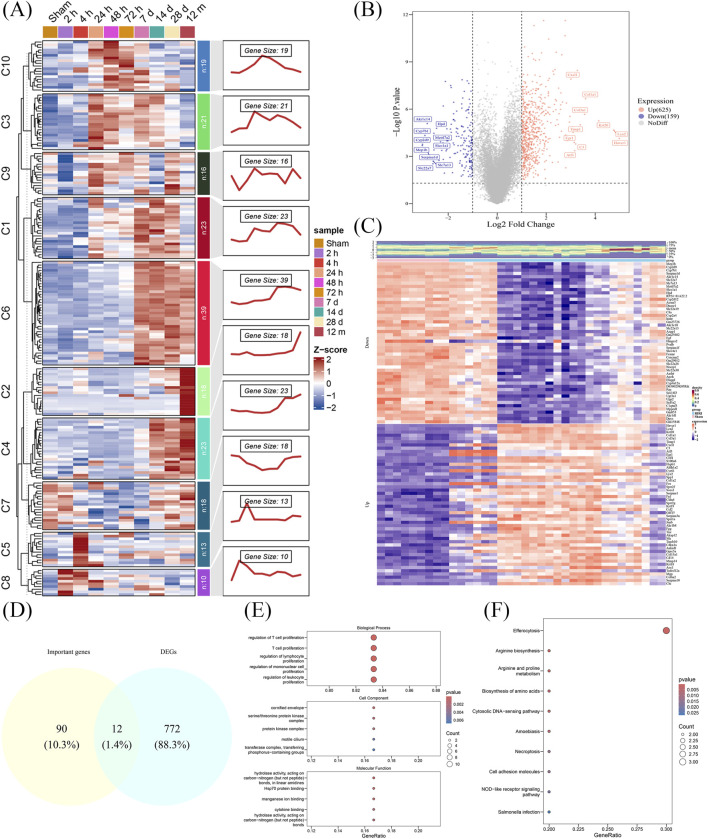
TRGs expression temporal dynamics in RIRI and identification of candidate genes. **(A)** Time-series clustering of TRGs in RIRI from 10 expression clusters. **(B,C)** A total of 784 DEGs were identified between RIRI and control samples. The top upregulated and downregulated genes were visualized in a volcano plot and heatmap. **(D)** Venn diagram showing 12 intersecting genes selected as candidate genes for further analysis. **(E,F)** Enrichment analysis of candidate genes.

To elucidate the expression dynamics of TRGs RIRI, a time-series analysis was conducted (Figure 1A). Early upregulated gene sets were prioritized for further investigation, which correlated with the rapid T cell proliferation response in RIRI. From clusters C1, C3, C5, C8, C9, and C10, 102 genes with an early upregulated tendency were identified. Then, a total of 784 DEGs were identified between the RIRI and Sham groups. The top 10 upregulated genes (e.g., *HAVCR1*, *LCN2*, *KRT20*) and downregulated genes (e.g., *MEP1B*, *CYP2D9*, *CYP7B1*) were visualized in volcano plots and heatmaps (Figures 1B,C; Supplementary Table S3). Through intersection analysis of DEGs and the 102 key genes, candidate genes were identified (Figure 1D).

To characterize the biological roles of candidate genes in RIRI, GO enrichment analysis identified 762 terms, including 697 BPs, 18 CCs, and 47 MFs (Supplementary Table S4). The top five BP terms included “T cell proliferation” and “regulation of leukocyte proliferation,” while CC terms featured “cornified envelope,” “protein kinase complex,” and “motile cilium.” For MFs, “Hsp70 protein binding” and “cytokine binding” were among the top terms (Figure 1E). Additionally, 10 KEGG pathways were enriched, including “efferocytosis,” “arginine biosynthesis,” and “amoebiasis” (Figure 1F; Supplementary Table S4). The insights gained from these findings are crucial for understanding the biological functions of genes related to RIRI advancement.

### 3.2 Identification of *ANXA1* and *ARG2* as key TRGs in IRI

**FIGURE 2:**
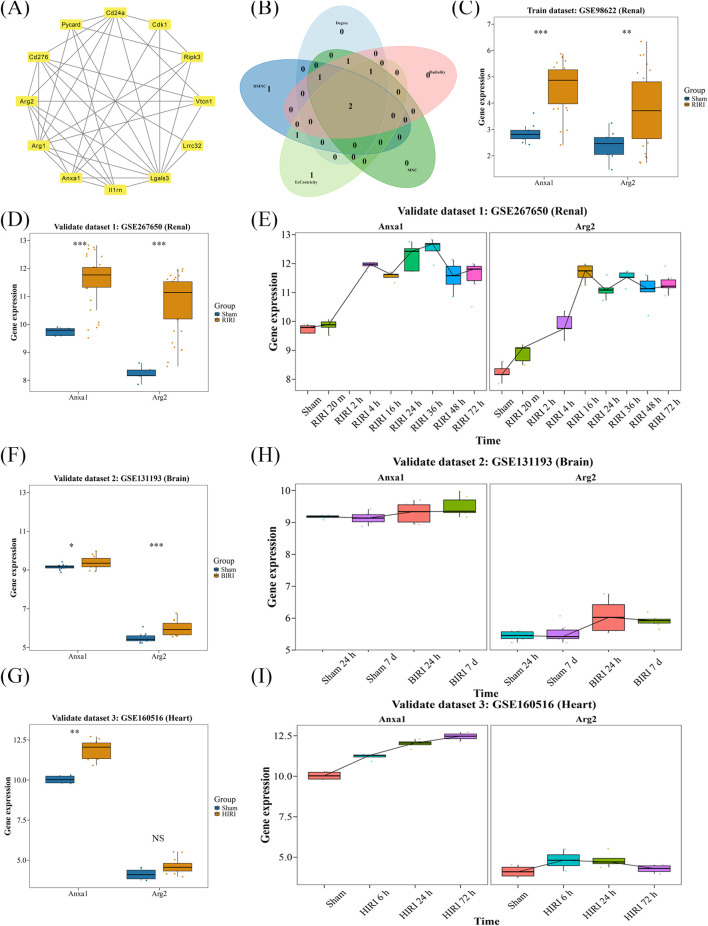
Identification and validation of key genes of TRGs in IRI. **(A)** PPI network analysis showed the connection of 12 candidate genes. **(B)** *ANXA1* and *ARG2* were selected as candidate key genes by intersecting the top five genes of five independent algorithms selection. **(C,D)** Both *ANXA1* and *ARG2* showed consistent and significantly upregulated expression in exploration and independent validation dataset. **(E)** Time-series expression showed both key genes exhibited early-stage upregulation in renal validation dataset. **(F,G)** In BIRI and HIRI validation datasets, *ANXA1* showed consistent upregulation; *ARG2* was only significant in BIRI. **(H,I)** Time trends confirmed *ANXA1* and *ARG2* elevation in BIRI and HIRI. *P < 0.05, **P < 0.01, ***P < 0.001.

To study the interactions among proteins encoded by candidate genes, a PPI network was created. Lgals3 displayed the highest connectivity, interacting with multiple genes, including *ANXA1*, *ARG2*, and *CDK1* (Figure 2A). *ANXA1* and *ARG2* were identified as key candidate genes through intersection analysis of the top five genes ranked by multiple algorithms (Figure 2B). Both genes exhibited consistent expression trends in the exploration and validation datasets for RIRI, with significant differences between the RIRI and Sham groups (Figures 2C,D). Consequently, *ANXA1* and *ARG2* were designated as key genes, showing upregulated expression in the early stages of RIRI in the validation dataset (Figure 2E). In HIRI and BIRI validation datasets, *ANXA1* expression was significantly different between groups, consistent with its trend in the exploration datasets. In contrast, *ARG2* showed significant intergroup differences only in BIRI (validation set 2), aligning with its trend in the training set, but not in the HIRI (validation set 3) (Figures 2F,G). Notably, *ANXA1* expression exhibited a consistent upward trend across all validation samples, whereas *ARG2* expression increased initially and then declined (Figures 2H,I). These findings highlight the complex pathogenesis of IRI and provide a clear foundation for further mechanistic studies and potential clinical translation.

### 3.3 The potential processes of key genes

**FIGURE 3:**
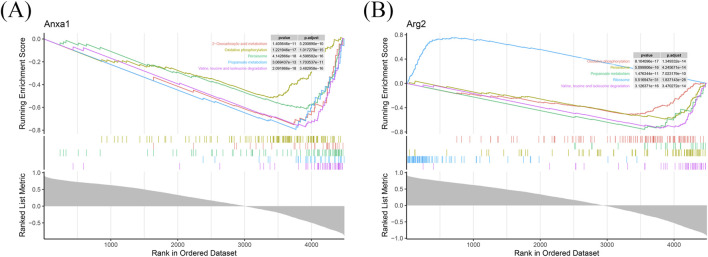
Functional annotation of *ANXA1* and *ARG2* in RIRI. GSEA identified 148 and 117 significantly enriched pathways for *ANXA1* and *ARG2*, respectively. **(A)** The top five pathways of ANX*A1* are shown based on the |NES|. **(B)** The top five pathways of *ARG2* are shown in RIRI. |NES|: absolute normalized enrichment score.

GSEA identified 148 and 117 pathways associated with *ANXA1* and *ARG2*, respectively (Supplementary Table S5). Enrichment score plots highlighted the top five enriched pathways for each gene, with “valine, leucine, and isoleucine degradation” ranking highest for both *ANXA1* and *ARG2* (Figures 3A,B). Notably, four of the top five pathways were co-enriched for both genes (Figures 3A,B), including oxidative phosphorylation, propanoate metabolism, peroxisome, and valine, leucine, and isoleucine degradation. This evidence provides a significant starting point for further clarifying the pathogenesis of IRI.

### 3.4 Immune cells infiltration and correlation with key genes

**FIGURE 4:**
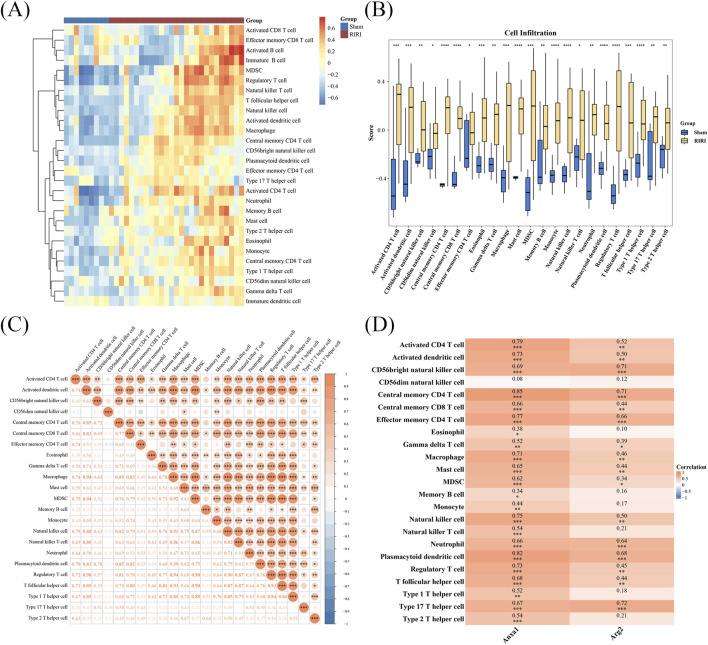
Immune infiltration and gene correlation analysis in RIRI. **(A,B)** A total of 23 immune cell types showed significant differences including 11 T cell subsets. The different cells were visualized in a heatmap and box plot. **(C)** Correlation analysis of different cell type **(D)** *ANXA1* strongly correlated with regulatory and central memory CD4^+^ T cells; *ARG2* correlated with Th17 and other T cell subsets, suggesting gene-specific T cell associations in IRI. *P < 0.05, **P < 0.01, ***P < 0.001.

Analysis of immune cell infiltration identified 23 immune cell types with significant differences between RIRI and Sham groups, including monocytes, central memory CD8 T cells, and mast cells (Figures 4A,B; Supplementary Table S6). Analysis of correlations indicated a strong positive link between activated dendritic cells and macrophages, and no significant negative correlations were detected among various immune cells (Figure 4C; Supplementary Table S6). Notably, *ANXA1* exhibited significant positive correlations with regulatory T cells, central memory CD4 T cells, and macrophages. And *ARG2* showed significant positive correlations with T helper 17 cells, plasmacytoid dendritic cells, and neutrophils (Figure 4D; Supplementary Table S5). These findings indicate that *ANXA1* and *ARG2* may drive RIRI pathogenesis by regulating specific immune cell populations, enhancing understanding of the underlying pathogenic mechanisms.

### 3.5 Drug prediction and target-drug interaction analysis for key genes in IRI

**FIGURE 5:**
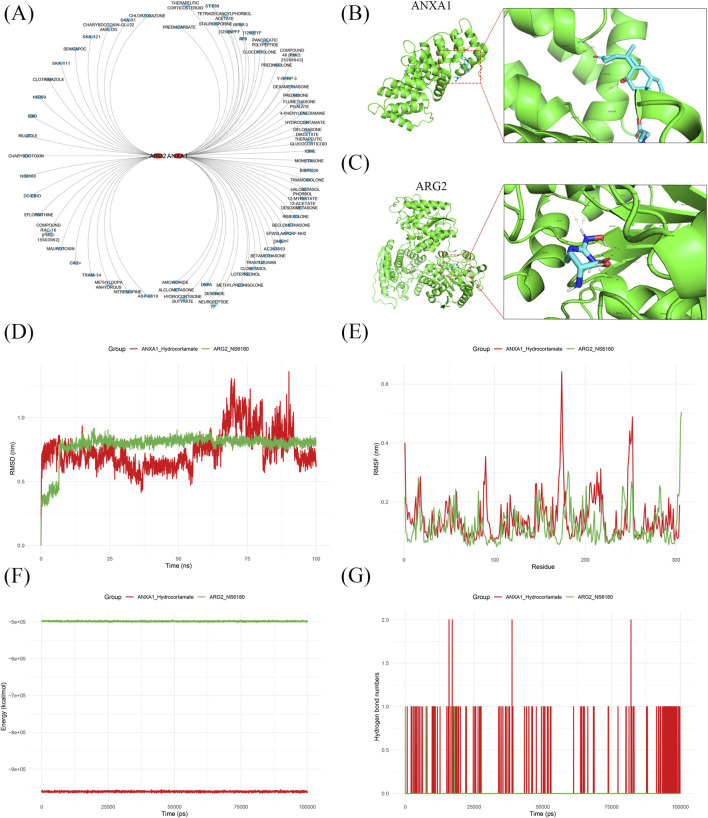
Drug prediction and target-drug interaction analysis for key genes in IRI. **(A)** 44 drugs targeting *ANXA1* and 21 targeting *ARG2* were predicted. **(B,C)** Molecular docking showed favorable binding energies for *ANXA1*-hydrocortamate (−3.96 kcal/mol) and *ARG2*-NS6180 (−4.74 kcal/mol). **(D,E)** Binding stability for *ANXA1*-hydrocortamate and *ARG2*-NS6180 analysis based on RMSD and RMSF fluctuations. **(F)** Energy analysis showed binding of *ANXA1* to hydrocortamate and *ARG2* to NS6180. **(G)** Hydrogen bond analysis confirmed the interactions target-drug complex.

A total of 44 *ANXA1*-related drugs (e.g., methylprednisolone, bibp3226, rimexolone) and 21 *ARG2*-related drugs (e.g., ebio, nitrendipine, ska-121) were identified (Figure 5A). Molecular docking analysis revealed that *ANXA1* bound to hydrocortamate with a binding energy of −3.96 kcal/mol, while *ARG2* exhibited a lower binding energy of −4.74 kcal/mol with NS6180 (Figures 5B,C; Supplementary Table S7). MDS was performed to further assess binding stability. The *ARG2*-NS6180 complex displayed smaller RMSD fluctuations compared to the *ANXA1*-hydrocortamate complex, indicating greater binding stability for *ARG2*-NS6180 (Figures 5D,E). Energy values differed significantly between the *ANXA1*-hydrocortamate and *ARG2*-NS6180 complexes throughout the simulation, with *ANXA1*-hydrocortamate showing tighter binding (Figure 5F). Additionally, the *ANXA1*-hydrocortamate complex exhibited the highest hydrogen bond density and intensity, reflecting stronger binding interactions (Figure 5G). These findings elucidate the interaction profiles of drugs with key gene receptor proteins, providing quantitative evidence and theoretical support for understanding drug-protein interaction mechanisms, predicting gene functions, and optimizing molecular models in RIRI.

### 3.6 Annotation of cell types in single-cell data

**FIGURE 6:**
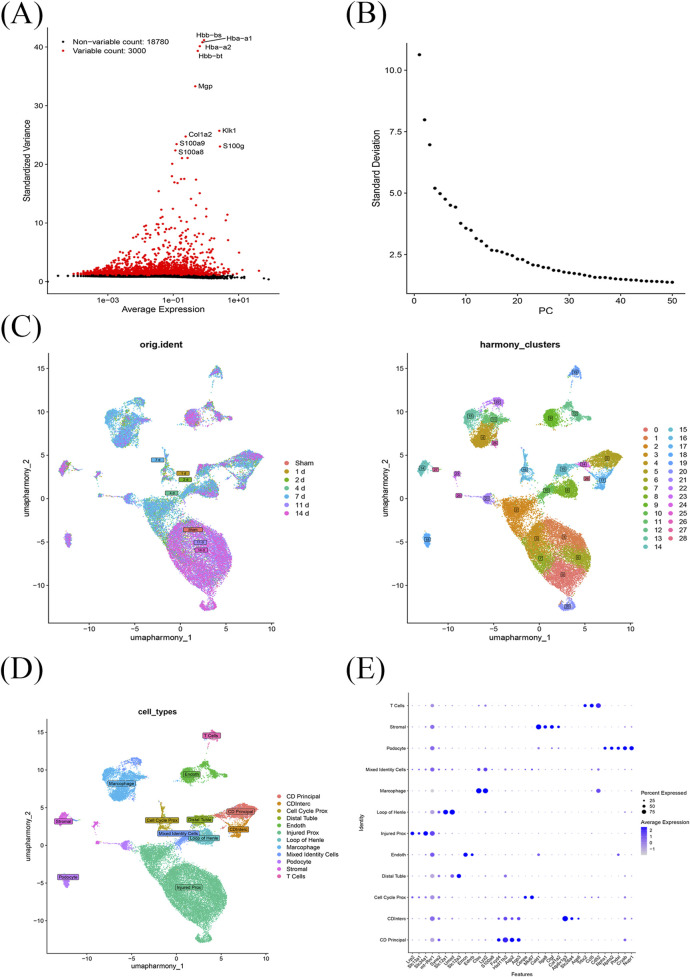
Single-cell transcriptomic profiling of RIRI. **(A)** A total of 21,780 genes from 33,337 cells across seven time points were obtained after quality control; the top 3,000 HVGs were selected. **(B,C)** Using the top 30 principal components, cells were clustered into 29 distinct groups. **(D)** These clusters were annotated into 12 major cell types. **(E)** Marker genes for cell type annotated.

Upon completing quality checks on the ScRNA sequencing data from the GSE139506 dataset, 21,780 genes across 33,337 cells were retained. The number of cells in the Sham, 1 day, 2 days, 4 days, 7 days, 11 days, and 14 days samples was 1,195, 1,834, 2,729, 5,204, 8,166, 9,211, and 4,998, respectively (Supplementary Figures S1–S2). Additionally, genes with high coefficients of variation across cells were extracted, and the top 3,000 HVGs were selected (Figure 6A). Using the top 30 principal components, samples were clustered into 29 distinct cell clusters (Figures 6B,C). Based on annotation methods, these clusters were classified into 12 major cell types: collecting duct principal cells, cell cycle proximal tubule cells, endothelial cells, injured proximal tubule cells, loop of Henle cells, macrophages, mixed identity cells, podocytes, stromal cells, collecting duct intercalated cells, distal tubule cells, and T cells (Figures 6D,E). These results provided a crucial theoretical basis for revealing the cellular pathogenic mechanism of RIRI, exploring potential genes and therapeutic targets.

### 3.7 T cell dynamics and cell communication in RIRI

**FIGURE 7:**
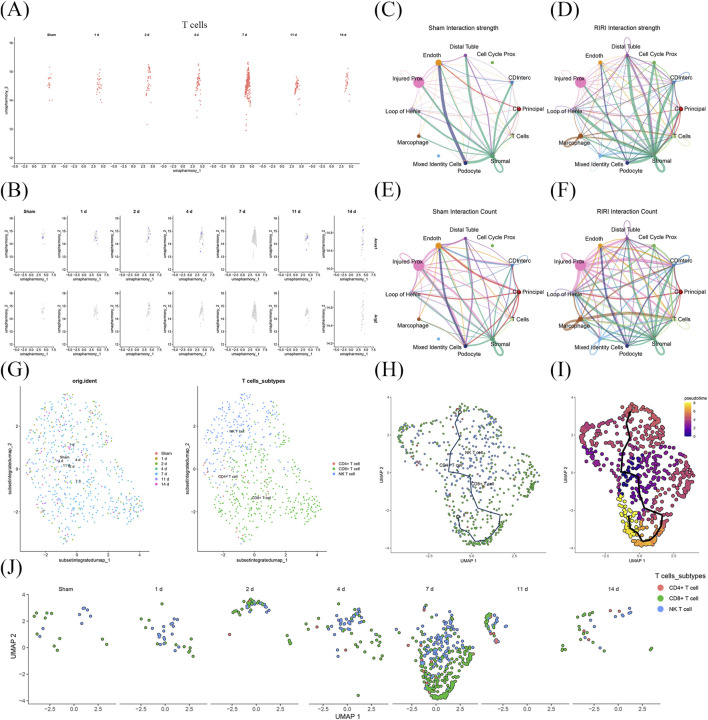
Stage-specific T cell dynamics and cellular interactions at single-cell transcriptomic level. **(A)** T cells showed a time-dependent increase followed by a decline post-RIRI. **(B)** *ANXA1* expression in T cells was stronger and more dynamic than *ARG2*, peaking outside day 7. **(C–F)** IRI enhanced more active cell–cell interactions, forming new communication patterns. T cells showed the strongest interaction with stromal cells, alongside notably strong and specific communication with macrophages. **(G–I)** CD4^+^ T cells dominated early, CD8^+^ T cells later, and NK T cells appeared mainly in early-to-middle phases. **(J)** T cell distributions became more clustered over time.

Time-course analysis of T cell alterations in RIRI revealed a trajectory of gradual increase followed by a decrease across various time points (Figure 7A). Notably, *ANXA1* exhibited more pronounced expression levels and distribution patterns in T cells compared to *ARG2*, with the lowest *ANXA1* expression observed at 7 days post-RIRI (Figure 7B). In the RIRI group, stromal cells showed robust interactions with multiple cell types, including loop of Henle cells and distal tubule cells, with T cell–stromal cell interactions being particularly strong (Figures 7C,D; Supplementary Figure S3). While in the Sham group, podocytes displayed significant interactions with endothelial cells, while T cells interacted only with endothelial and stromal cells (Figures 7E,F; Supplementary Figure S4). Ligand-receptor analysis in the RIRI group identified *Spp1-(Itga8+Itgb1)* as the strongest interaction when T cells acted as signal receivers from stromal cells, and *Ptn-Ncl* as the strongest when T cells were signal senders to stromal cells. In the Sham group, *Gzma-F2r* was the dominant interaction for T cells as signal receivers, while *Ptn-Ncl* remained the strongest for T cells as signal senders (Supplementary Figures S5–S6). T cells were further classified into three subtypes: CD4^+^ T cells, CD8^+^ T cells, and natural killer T (NKT) cells (Figure 7G). CD4^+^ T cells predominated in the mid-early stage, CD8^+^ T cells in the late stage, and NKT cells in the early and middle stages (Figures 7H,I). Initially, CD8^+^ T cells and NKT cells were widely distributed in the Sham group, but in RIRI, they became more concentrated, notably at the 7-day mark (Figure 7J). These findings provide a mechanistic basis for understanding RIRI pathophysiology and highlight potential therapeutic targets for intervention.

### 3.8 Validation of T cell proliferation and expression of *ANXA1* and *ARG2* in RIRI

**FIGURE 8:**
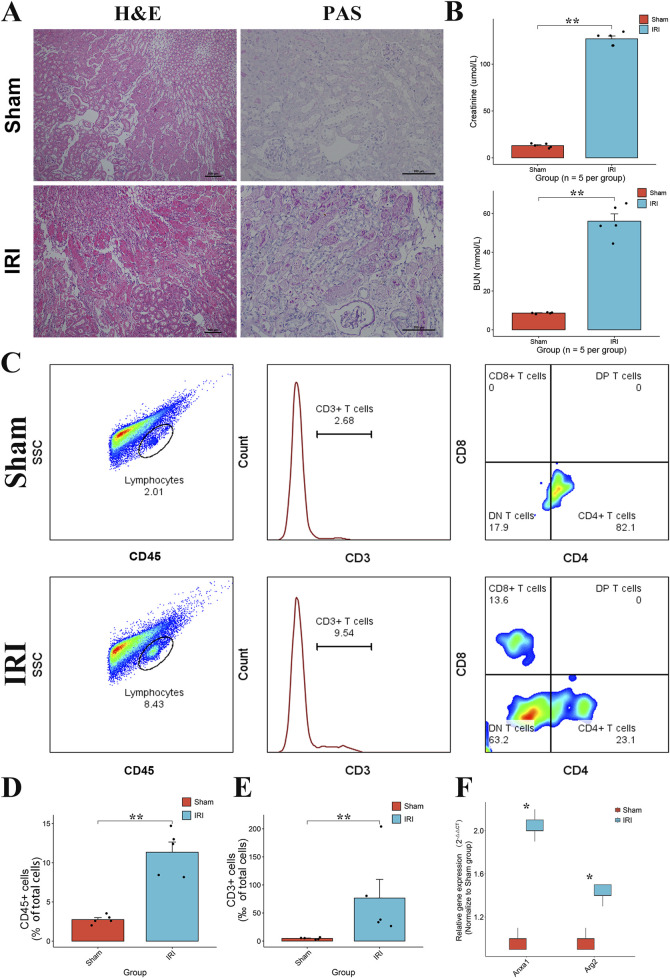
Validation of T cell proliferation and the expression of *ANXA1* and *ARG2* in RIRI. **(A)** Representative H&E and PAS staining of kidney tissues from sham and IRI mice showing tubular damage in the IRI group. **(B)** Serum creatinine and BUN levels were significantly elevated in IRI mice, indicating impaired renal function. **(C)** Analysis of immune cells in the kidney after IRI. The gating strategy identifies total leukocytes (CD45^+^), T cells (CD3^+^), and their CD4^+^ and CD8^+^ subsets. **(D,E)** The numbers of these cells increased in the injured kidneys. **(F)** Relative mRNA expression levels of *ANXA1* and *ARG2* in the kidney tissues, measured by RT-qPCR, showing significant upregulation in the IRI group. Data are presented as mean ± SD. *P < 0.05, **P < 0.01, ***P < 0.001. H&E, hematoxylin and eosin; PAS, periodic acid–Schiff.

To validate the T cell activities and the expression of key genes, we constructed the bilateral RIRI mice as previously described. Compared to the sham group, the RIRI group exhibited notable tubular injury as revealed by H&E and PAS histological staining (Figure 8A). Serum creatinine and blood urea nitrogen levels were significantly elevated, confirming renal dysfunction (Figure 8B). Flow cytometric analysis showed a marked increase in lymphocyte infiltration, with significantly higher proportions of CD45^+^ leukocytes and CD3^+^ T cells in IRI kidneys (Figures 8C–E). Among CD3^+^ T cells, CD4^+^ subsets were predominant, accompanied by a relative increase in CD8^+^ T cells. qPCR analysis further demonstrated significant upregulation of *ANXA1* and *ARG2* expression in the RIRI group (Figure 8F), supporting their involvement in early T cell–mediated immune responses.

## 4 Discussion

IRI remains a significant clinical challenge, with early immune responses contributing substantially to tissue damage (Zhang M. et al., 2024). Although T cells are recognized as key contributors to IRI pathogenesis, the mechanisms governing their rapid activation and proliferation remain incompletely understood. The heterogeneity of T cell subsets and their stage-specific infiltration complicate efforts to define their precise roles in IRI (Lee and Jang, 2022). Integrating single-cell and bulk RNA sequencing data, this study systematically characterized the temporal dynamics of T cell proliferation in RIRI. *ANXA1* and *ARG2* were identified and validated as key TRGs, with hydrocortamate and NS6180 emerging as potential therapeutic agents targeting these genes. Early infiltration of diverse T cell subsets, including CD4^+^, CD8^+^, and NKT cells, was observed, consistent with dynamic regulation in IRI. The discoveries shed light on the immune microenvironment and transcriptional networks linked to IRI, suggesting that targeting T cells could be a promising strategy for developing therapies.

TRGs exhibit distinct temporal regulation during IRI. The expression profiles of TPGs vary across time points, abundance clusters display progressively increasing tendencies in the different phases, indicating predominant activation and sustained involvement in IRI development. Early T cell proliferation correlates with exacerbated tissue damage, and the depletion of T cells attenuates tissue damage in IRI (Lai et al., 2007; Martín and Sánchez-Madrid, 2025), while later proliferation may contribute to chronic pathological changes like fibrosis (Xu et al., 2022), potentially driven by distinct T cell subset distributions. This study focuses on T cell proliferation in the acute phase, so we analyzed the early upregulated gene cluster. Functional annotation of the candidate genes supports their central role in mediating T cell-driven immune responses. *ANXA1* and *ARG2* emerged as key hub genes with potential regulatory significance, identified through multiple independent computational algorithms, highlighting their robust candidacy as therapeutic targets.

*ANXA1*, traditionally recognized as an anti-inflammatory mediator in innate immunity (Wu et al., 2021), was identified as a key gene strongly associated with T cell activity, indicating a dual role in immune regulation. The role of ANXA1 is multifaceted in adaptive immunity. ANXA1 can be secreted by T cells and critically shapes early immune responses by promoting regulatory T cell differentiation, enhancing Th1 and Th17 development, and suppressing Th2 polarization (Gavins and Hickey, 2012). These effects contribute to pro-inflammatory phenotypes during the progression of inflammatory diseases such as rheumatoid arthritis and primary sclerosing cholangitis (Kelly et al., 2022; Zhang et al., 2023). Notably, excessive inflammatory conditions may reverse its regulatory influence on T cells (Gavins and Hickey, 2012). Emerging evidence from tumor microenvironments further demonstrates that ANXA1 modulates the maturation of dendritic cells and macrophages, leading to reduced T cell activation and immune evasion (Zhang et al., 2024c; Jiang et al., 2025). Given its diverse roles in immune regulation, the function of ANXA1 in IRI extends beyond mere biomarker status. The role of *ANXA1* in early T cell proliferation expands our understanding of adaptive immune mechanisms in IRI and provides valuable clues for future mechanistic studies and potential immunomodulatory interventions.

*ARG2* is identified in this study as an emerging regulator of T cell–mediated responses during IRI. Correlation analysis showed the strongest association with Th17 cells, indicating a potential role in pro-inflammatory T cell subsets. Prior studies have revealed that *ARG2* accumulation in tubular cells during AKI (Zhou et al., 2023), where its overexpression promotes nitrosative stress and apoptosis. However, its function in T cell-mediated injury remains uncharacterized in IRI. *ARG2* expression has also been associated with immune cell infiltration in inflammatory and autoimmune diseases, like steroid-induced osteonecrosis (Yu et al., 2021), supporting its broader immunomodulatory relevance. However, the arginine metabolism presents a controversial role in immune regulation in different disease models, exhibiting both promoting and inhibitory roles in T cell activation (Asosingh et al., 2020; Starikova et al., 2023). *ARG2* may act as a metabolic checkpoint in T cells and innate kidney tissue during RIRI, with implications for therapeutic modulation of both immune and non-immune responses.

Hydrocortamate and NS6180 were identified as potential therapeutic agents targeting T cell proliferation in IRI, through a comprehensive drug discovery strategy *in silico* based on the key regulatory genes. Hydrocortamate is a synthetic glucocorticoid with confirmed anti-inflammatory effects (Zheng et al., 2015). *ANXA1*, a known glucocorticoid-responsive gene (Patel et al., 2012), may mediate this effect by bridging steroid signaling and immune modulation in IRI, as prior studies have confirmed the efficacy of glucocorticoids in mitigating inflammation in IRI (Kumar et al., 2009; Escudero et al., 2024). NS6180, a selective KCa3.1 channel inhibitor (Brown et al., 2018), targets T cell activation and proliferation with high specificity. It exhibits potent efficacy in restricting T cell-driven inflammation (Strøbæk et al., 2013). Along with a newly observed link to *ARG2*, NS6180 may also modulate T cell metabolism and enhance therapeutic potential in IRI. These findings position Hydrocortamate as a broad immunosuppressive agent with notable T cell regulatory effects, and NS6180 as a targeted T cell inhibitor, offering complementary therapeutic strategies for IRI. Additional studies, both preclinical and clinical, are needed to validate their safety and effectiveness in the treatment of IRI.

T cells exhibit dynamic infiltration and activation patterns in IRI, with their numbers rapidly increasing early in the injury process and subsequently declining, reflecting stage-specific regulation. Integrated transcriptomic analyses revealed diverse CD4^+^ T cell subsets, including central memory CD4^+^ T cells, Treg, Th1, Th2, and Th17 cells, each contributing uniquely to RIRI pathophysiology. Central memory CD4^+^ T cells, distinguished by their differentiation state and regenerative capacity, may contribute to sustained injury in RIRI (Ascon et al., 2009). Tregs mitigate acute injury and promote repair in early RIRI by suppressing excessive inflammation (Kinsey et al., 2010). Conversely, Th17 cells exacerbate kidney damage in the acute phase by secreting pro-inflammatory cytokines (Mehrotra et al., 2020), though they may contribute to repair in later stages. NKT cells, proliferating early, likely exert protective effects by attenuating regional tissue damage (Zhang et al., 2014; Tamura et al., 2024). CD8^+^ T cells, exhibiting delayed proliferation, may contribute to chronic kidney damage (Xu et al., 2022; Jiang et al., 2024). ANXA1 promotes Th1 differentiation by upregulating T-bet and IFN-γ, whereas its effects on Th17 cells involve restricting IL-17 in Th17-associated diseases such as uveitis, yet promoting Th17 accumulation in other conditions like experimental autoimmune encephalomyelitis (Kelly et al., 2022). In IRI, ANXA1 may exert bidirectional effects on T cell subsets: it fosters inflammatory injury while preserving the potential to limit excessive Th17-driven responses and promote inflammation resolution. Beyond directly modulating functional T cell subsets such as Tregs, ARG2—which metabolizes arginine—may exert a more universal regulatory role. It not only modulates T cell activity but also influences other immune components like macrophages (Lowe et al., 2019; Zhu et al., 2025). Furthermore, T cells respond to macrophage-derived chemokines, coordinating immune responses within the inflammatory microenvironment and highlighting their synergistic role with macrophages (Rao et al., 2014). Collectively, these findings underscore the multifaceted roles of T cells in mediating both injury and repair in RIRI and offer new perspectives for developing immunomodulatory therapies targeting specific T cell subsets.

This study identified key genes associated with T cell proliferation in ischemia-reperfusion injury and provided initial insights into their potential roles, highlighting novel targets for early therapeutic intervention. T cells represent a highly diverse family with distinct roles across different stages of IRI. The exploration of the regulatory mechanisms driving T cell activation and proliferation, as well as the functional heterogeneity of specific T cell subsets, remains incomplete. At the same time, murine T cell responses may not fully mirror human IRI, limiting direct clinical extrapolation. Moreover, beyond the transcriptomic data, future studies could integrate multi-omics methods, including proteomics, metabolomics, and spatial transcriptomics approaches to validate and expand these findings, achieving a more comprehensive understanding of T cell-mediated immune responses in IRI. Subsequent research could also further explore specific T cell subsets (such as Th1, Th17, and Treg) or key molecular drivers to deepen mechanistic insights. While early T cell activation is evident in IRI, there is a lack of sufficient temporal resolution to capture the earliest regulatory events. Shifting the analytical focus from days to hours may provide more nuanced insights into immune dynamics. Overall, our findings suggest that TRGs contribute to the rapid T cell response in IRI, with multiple T cell subsets dynamically participating in early injury progression. *ANXA1* and *ARG2* emerged as representative regulatory drivers and potential therapeutic targets, offering new perspectives for precision treatment in IRI.

## Data Availability

The datasets presented in this study can be found in online repositories. The names of the repository/repositories and accession number(s) can be found in the article/Supplementary Material.

## Abbreviations

AKI, acute kidney injury; BIRI, brain ischemia-reperfusion injury; BUN, blood urea nitrogen; CC, cellular components; DEGs, differentially expressed genes; DGIdb, Drug-Gene Interaction Database; GO, Gene Ontology; GSEA, gene set enrichment analysis; H&E, Hematoxylin and Eosin; HIRI, heart ischemia-reperfusion injury; HVGs, highly variable genes; IRI, ischemia-reperfusion injury; KEGG, Kyoto Encyclopedia of Genes and Genomes; log2FC, log2 fold change; MDS, molecular dynamics simulation; MF, molecular functions; MVB, multivesicular body; NES, absolute normalized enrichment score; NKT cell, natural killer T cell; PAS, Periodic Acid-Schiff; PCA, principal component analysis; RIRI, renal ischemia-reperfusion injury; RMSD, root-mean-square deviation; RMSF, root-mean-square fluctuation; sCr, serum creatinine; ScRNA, Single-cell RNA; TRGs, T cell proliferation–related genes; UMAP, uniform manifold approximation and projection.
