# Supplementary material for: ANXA1 and ARG2 drive T cell proliferation in ischemia-reperfusion injury: integrated bulk and single-cell transcriptomic analysis
Source: Front Cell Dev Biol. 2025 Sep 4;13:1673163. doi: 10.3389/fcell.2025.1673163 (PMC12443811; doi:10.3389/fcell.2025.1673163)
Supplement: Supplementary file 4 [file DataSheet1.pdf]

Supplementary material

Anxa1 and Arg2 Drive T Cell Proliferation in Ischemia-Reperfusion Injury: Integrated Bulk and Single-Cell Transcriptomic Analysis

Haofeng Zheng#, Kaiming He#, Jianchao Wei#, Wangtianxu Zhou, Zhiyi Kong, Qingfu Dai, Jieyi Dong, Zihuan Luo, and Qiquan Sun, MD, PhD

Department of Renal Transplantation, Guangdong Provincial People's Hospital (Guangdong Academy of Medical Sciences), Southern Medical University, Guangzhou, China

Brief title: Role of T cell proliferation in IRI

Correspondence author: Dr Qiquan Sun, 106, 2nd road Zhongshan, Yuexiu District, Guangzhou, Guangdong, China, 510080, Email: sunqiquan@gdph.org.cn; Tel. +86 138 2510 9488

#These authors contributed equally to this work.

This article contains the following supplemental material

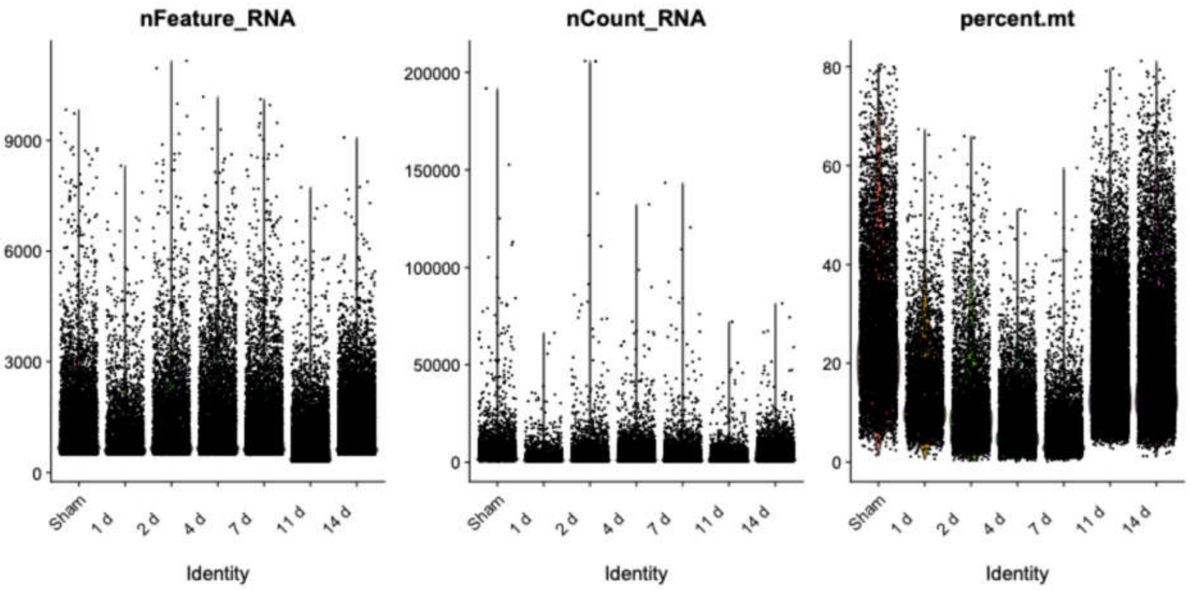

Supplemental Figure 1. Quality control for single-cell RNA sequencing before filtrating. (A) the number of unique genes detected in a single cell. (B) the total number of RNA molecules detected in a single cell. (C) the percentage of mitochondrial gene expression out of the total RNA counts

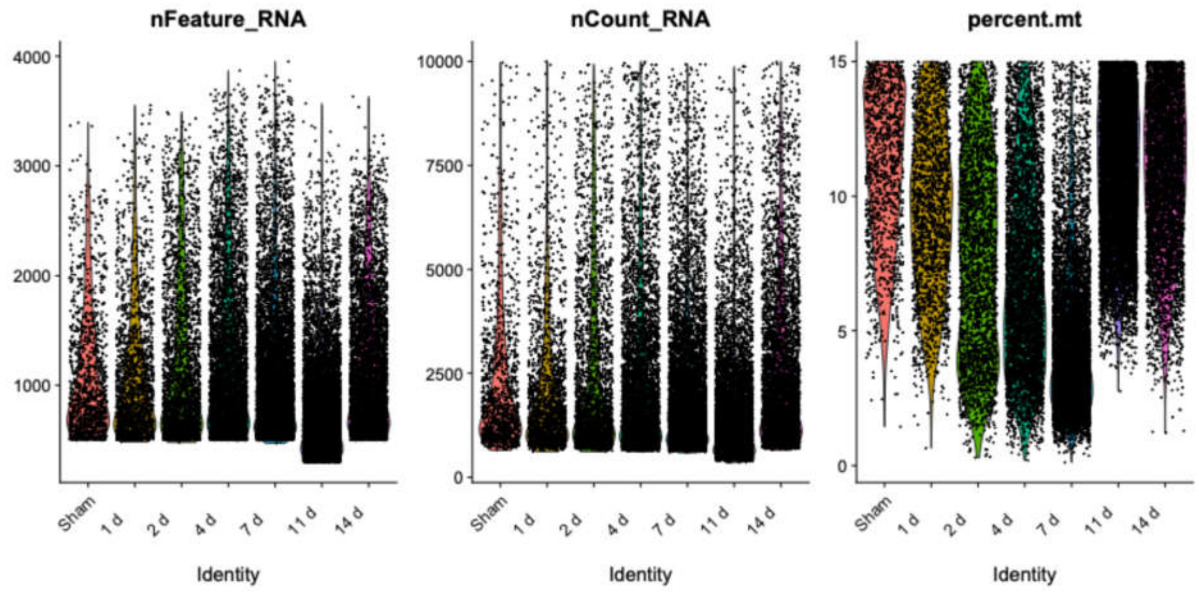

Supplemental Figure 2. Quality control for single-cell RNA sequencing after filtrating. (A) the number of unique genes detected in a single cell. (B) the total number of RNA molecules detected in a single cell. (C) the percentage of mitochondrial gene expression out of the total RNA counts

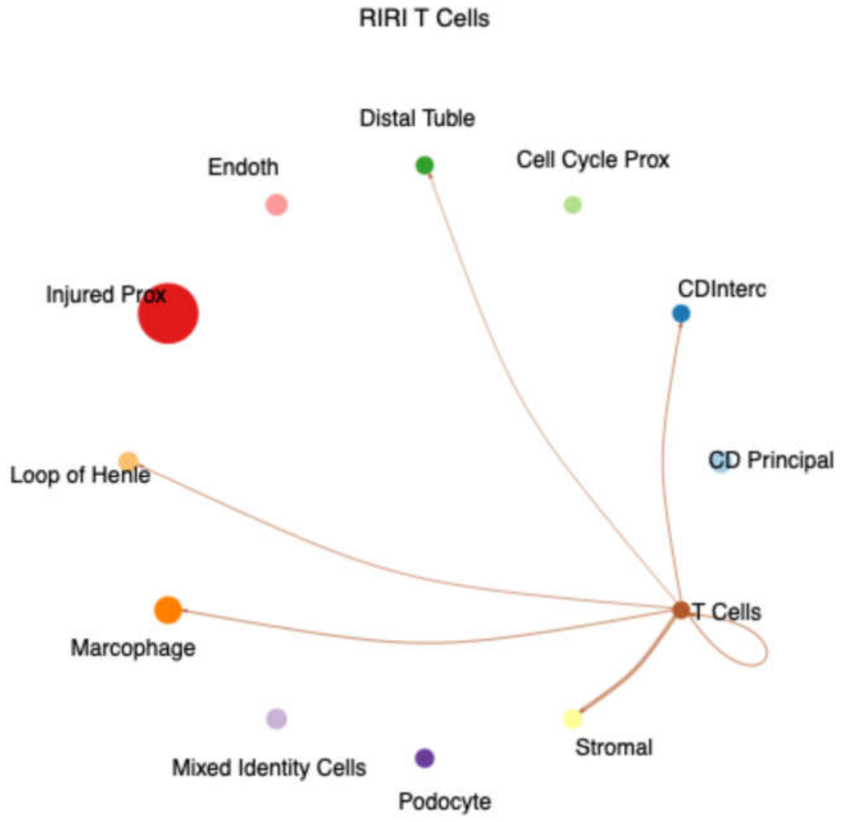

Supplemental Figure 3. T cell as an active role in cell communication network in in RIRI. T cells exhibit intercellular communication with stromal cells, loop of Henle cells, macrophages, distal tubule cells, and CD intercalated cells, highlighting their active role in orchestrating immune responses and tissue remodeling during RIRI.

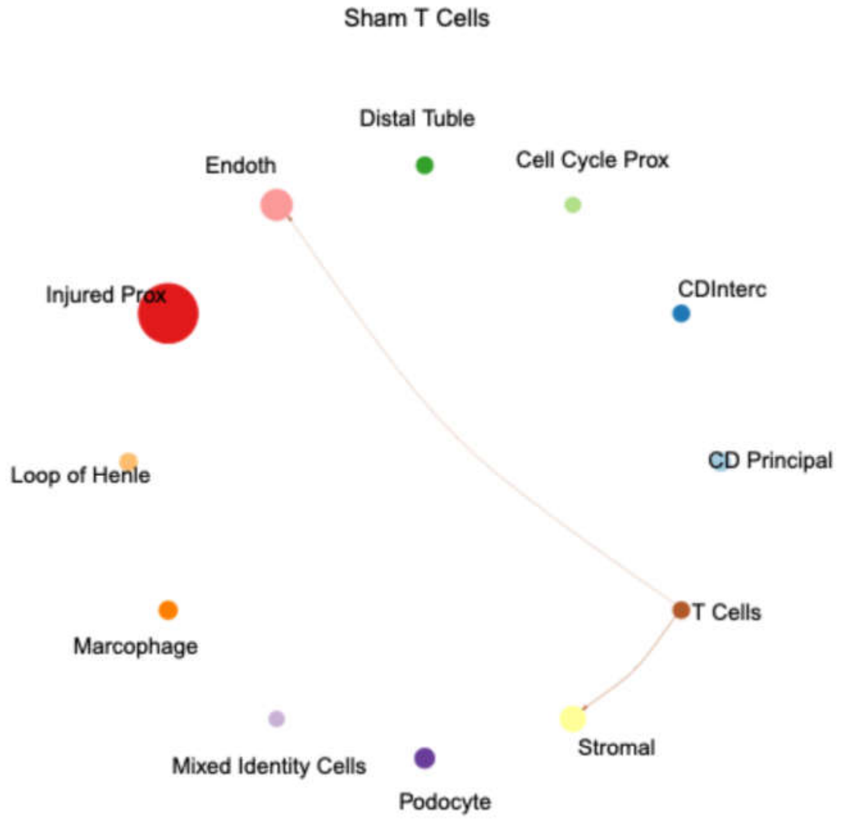

Supplemental Figure 4. T cell communication network in Sham group. T cells exhibit limited communication in non-injured kidney microenvironment. Cellular interactions were only observed between T cells and stromal cells, as well as endothelial cells, indicating a quiescent immune state in the absence of ischemic injury.

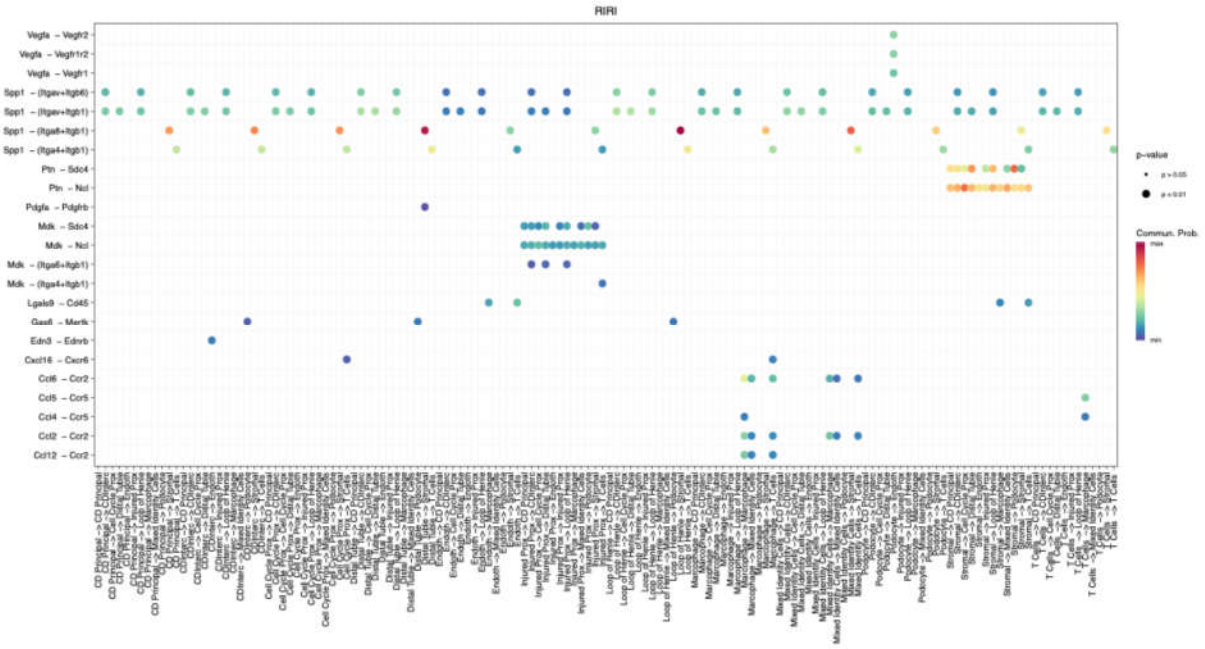

Supplemental Figure 5. Overview of the ligand-receptor interactions of cells in RIRI. Ligand-receptor signaling axes interactions of cell in RIRI, showing the active cells communication in RIRI. Notably, the communication probability of the Ptn-Ncl, Spp1 − (Itgav+Itgb6), Spp1 − (Itgav+Itgb1) pair is significantly higher in RIRI samples. The color represents the expression level of interactions intensity; the size represents the p value of interactions.

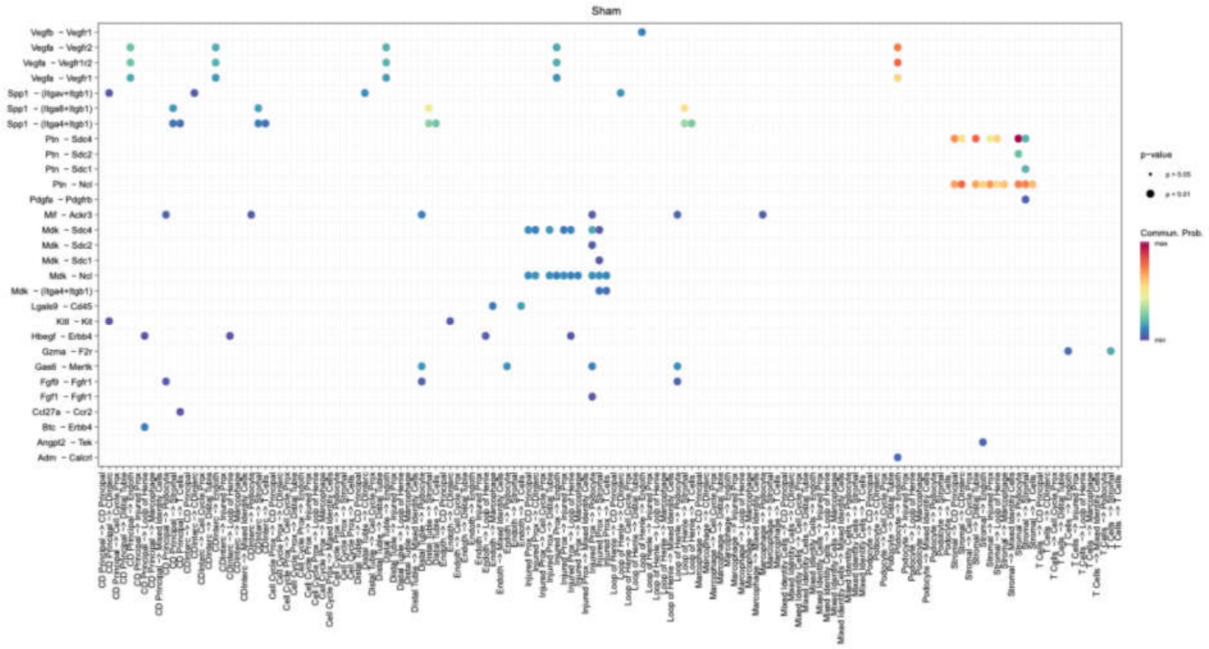

Supplemental Figure 6. Overview of the ligand-receptor interactions of cells in Sham group. Ligand-receptor signaling axes interactions of cell in sham group. The color represents the expression level of interactions intensity; the size represents the p value of interactions.

Supplemental Table Legend

Supplementary Table 1. T Cell Proliferation Related Genes List.

Supplementary Table 2. Primer sequences for Real-time quantitative PCR.

Supplementary Table 3. Differentially Expressed Genes of Renal Ischemia-Reperfusion Injury

Supplementary Table 4. Enriched Terms List of Candidate Genes in GO and KEGG

Supplementary Table 5. Gene Set Enrichment Analysis of Key Genes in Renal Ischemia-Reperfusion Injury

Supplementary Table 6. Immune Cells Infiltration Proportion and Correlation with Key Genes

Supplementary Table 7. Molecular Docking Results of Anxa1-Hydrocortamate and Arg2- NS6180
